# Supplementary material for: Tracing the influence of land-use change on water quality and coral reefs using a Bayesian model
Source: Sci Rep. 2017 Jul 6;7:4740. doi: 10.1038/s41598-017-05031-7 (PMC5500483; doi:10.1038/s41598-017-05031-7)
Supplement: Supplementary file 1 — Supplementary material [file 41598_2017_5031_MOESM1_ESM.pdf]

Tracing the influence of land-use change on water quality and coral reefs using a Bayesian model

Christopher J. Brown[1,*] Stacy D. Jupiter[2], Simon Albert[3], Carissa J. Klein[4], Sangeeta Mangubhai[2], Joseph M. Maina[5,6], Peter Mumby[7], Jon Olley[1], Ben Stewart-Koster[1], Vivitskaia Tulloch[6], Amelia Wenger[8]

1. Australian Rivers Institute, Griffith University, Nathan, Queensland, 4111, Australia;

2. Wildlife Conservation Society, Melanesia Program, 11 Ma'afu Street Suva, Fiji;

3. School of Civil Engineering, The University of Queensland, Queensland, 4072, Australia

4. Geography, Planning, and Environmental Management, The University of Queensland, Queensland, 4072, Australia

5. Department of Environmental Sciences, Macquarie University, Sydney, Australia

6. Australian Research Council Centre of Excellence for Environment Decisions, Centre for Biodiversity and Conservation Science, Department of Biological Sciences, The University of Queensland, Brisbane, Australia

7. Marine Spatial Ecology Laboratory School of Biological Sciences, Goddard Building, The University of Queensland, Brisbane, QLD 4072, Australia.

8. ARC Centre of Excellence for Coral Reef Studies, James Cook University, Townsville, QLD, Australia, 4811

*To whom correspondence should be addressed: chris.brown@griffith.edu.au

Supplementary material overview

Code for the Bayesian models (Appendix A); Parameter settings for the MCMC chain (Appendix B) methods for mapping land-cover and calculating sediment run-off (Appendix C); methods for benthic habitat models (Appendix D); details of the simulation study (Appendix E); turbidity data used to fit Bayesian model (Appendix F); benthic habitat survey data (Appendix G)

Supplementary material

Appendix A: Model code

See attached file

Appendix B: Parameter settings for the fitting the Bayesian model to the Vanua Levu case-study

Table B1 Specification of prior distributions

**Parameter**	**Prior distribution**	**Parameters**
τ_y	Gamma	Rate = 0.001, scale = 0.001
τ_υ	Gamma	Rate = 0.001, scale = 0.001
α_j	Independent gamma for each coast	Rate = 0.001, scale = 0.001
θ	Log-normal	Mean = 0, precision = 0.001

Table B2 Parameter settings for the MCMC simulations. Multiple values indicate values used for different chains.

**Parameter**	**Values**
Number of chains	3
Burn-in samples	5000
Number of samples	18000
Thinning	15
Initial values for α	1.38
Initial values for τ_y	2
Initial values for τ_υ	2.3, 4.6, 1.75
Initial values for θ	6.4, 3.2, 1.6

Appendix C: Identification of catchments for Vanua Levu, land-cover and calculation of rainfall run-off

Delineation of catchments

Catchments were delineated using the SRTM DEM (1 arc-second resolution [1]) and the 'raster watershed' routine in GRASS GIS, implemented from the R programming environment [2,3]. Following catchment delineation we used the 'raster water outlet' routine in GRASS GIS to identify catchment river mouths. We then manually corrected catchments and river mouths to

reposition digitally located river mouths on actual river mouths, as identified using Google Earth. The final map had 74 catchments for the study region.

Calculation of sediment loads

Land-cover in each catchment was estimated using Landsat 8 images [4] and object-based image analysis (Appendix S2). Each terrestrial pixel (30 by 30 metre resolution) was allocated to vegetated or non-vegetated classes. We then use an interpolated rainfall map, provided by the Wildlife Conservation Society, to calculate wet-season rainfall for each catchment, spatially averaging across all pixels in a catchment to obtain a catchment's average rainfall (in mm).

Rainfall run-off was calculated as the difference between rainfall and predicted evapotranspiration. Evapotranspiration was estimated using functions from a meta-analysis of evapotranspiration across catchments with different rainfalls [5]. It was reasonable to ignore soil water and ground-water recharge in the water balance, because we are interested in long-term equilibrium dynamics [5]. Thus, the proportion of rainfall running off each land-use type in a given catchment was estimated:

$$p_{l,k} = 1 - E_{k,l}$$

Where $E_{k,l}$ is the proportion of evapotranspiration and $r'_{k,l}$ is rainfall in mm. Evapotranspiration was estimated:

$$E_{k,l} = \frac{\left(1 + w\left(\frac{E_0}{r'_{k,l}}\right)\right)}{\left(1 + w\left(\frac{E_0}{r'_{k,l}}\right) + \left(\frac{r'_{k,l}}{E_0}\right)\right)}$$

Where *E0* and *w* are given by Zhang, et al. [5]. E_0 = 1410 or 1100 for forest or grassland respectively and w = 2 or 0.5 for forest and grassland respectively.

Sediment yields for vegetated and un-vegetated land-uses (equation 4 main text) were taken from a review of northern monsoon catchments in the Great Barrier Reef, where sediment yield in natural catchments was 32 mg/L and for degraded catchments was 99 mg/L (Neil et al. 2002).

We also applied this model at resolution of the land-use data (1-arc second) and aggregated values across catchments, however results were nearly identical toe the catchment scale calculations (r^2 = 0.99), so we present the simpler catchment scale results here.

Appendix D: benthic habitat models

We used linear models for each habitat type that were appropriate for the distribution of residuals. For algae and silt cover there were a high proportion of zero observations, so we fit fit hurdle models, with a binomial phase for the presence of silt/algae and normal phase for positive values of silt/algae cover [6]. Positive values of silt cover were log transformed and positive values of algae cover logit transformed to attain normality. A significant effect of water quality on the binomial phase indicates the level at which poor water quality results in sediment settlement or algae outcompeting other benthic species. A significant effect of water quality on the normal phase indicates the rate at which silt or algal cover increases with poor water quality.

Proportion cover of algae and coral models did not satisfy the assumption of normal residuals. Therefore, we transformed proportion cover using the logit transform, which normalized

residuals (Warton and Hui 2011). For all models we obtained 95% confidence limits using the non-parametric resampling[7].

Coral genera that were sediment sensitive were classified based on existing literature for Indo-Pacific corals and included: *Acropora*, *Montipora*, *Astreopora*, *Pocillopora*, *Favia* [8-12].

Appendix E: Simulation study

Calculation of fit statistics for the simulation study

The relative error for a single random data-set was calculated

$$R^j(\theta) = \left(med\left(\hat{\theta} | Y^j\right) - \theta\right) / \theta$$

Where, $med(\hat{\theta} | Y^j)$ is the median estimate of the parameter, given the jth simulated data-set $\mathbf{Y}^j$ and θ is the true value of the parameter. The mean relative error was then estimated as the mean of $R^j(\theta)$ across J=25 random data-sets.

The coefficient of variation for each simulation trial was calculated

$$CV(\theta) = sd\left(\hat{\theta} | Y^j\right) / med\left(\hat{\theta} | Y^j\right)$$

Where $sd(\hat{\theta} | Y^j)$ is the standard deviation of the parameter estimate for the jth trial. Again, we took the mean across J trials to estimate the mean coefficient of variation.

Table E1: Parameter values for the simulation test study.

**Parameter**	**Values**
Number of chains	3
Burn-in samples	5000
Number of samples	15000
Thinning	15
Initial values for α	1.38
Initial values for τ_y	2

Table E2: Parameter settings for MCMC chains in the simulation test study

**Parameter**	**Values**
Number of chains	1
Burn-in samples	1000
Number of samples	10000
Thinning	5

References

1 Farr, T. G. *et al.* The shuttle radar topography mission. *Reviews of geophysics* **45** (2007).
2 GRASS Development Team. Geographic Resources Analysis Support System (GRASS) Software, Version 6.4.4. (Open Source Geospatial Foundation, 2014).
3 Bivand, R. spgrass6: Interface between GRASS 6 and R. R package version 0.8-6 (2007). https://CRAN.R-project.org/package=spgrass6
4 Roy, D. P. *et al.* Landsat-8: Science and product vision for terrestrial global change research. *Remote Sensing of Environment* **145**, 154-172, doi:http://dx.doi.org/10.1016/j.rse.2014.02.001 (2014).
5 Zhang, L., Dawes, W. & Walker, G. Response of mean annual evapotranspiration to vegetation changes at catchment scale. *Water resources research* **37**, 701-708 (2001).
6 Zuur, A., Ieno, E., Walker, N., Saveliev, A. & Smith, G. *Mixed Effects Models and Extensions in Ecology with R*. (Springer, 2009).
7 Davison, A. C. & Hinkley, D. V. *Boostrap Methods and Their Applications*. (Cambridge University Press, 1997).
8 Perry, C. T. & Smithers, S. G. Taphonomic signatures of turbid-zone reef development: examples from Paluma Shoals and Lugger Shoal, inshore central Great Barrier Reef, Australia. *Palaeogeography, Palaeoclimatology, Palaeoecology* **242**, 1-20 (2006).
9 Vermaatl, J. E. Damage and recovery of four Philippine corals from short-term sediment burial. *Mar Ecol Prog Ser* **176**, 11-15 (1999).
10 Fabricius, K., De'ath, G., McCook, L., Turak, E. & Williams, D. M. Changes in algal, coral and fish assemblages along water quality gradients on the inshore Great Barrier Reef. *Marine pollution bulletin* **51**, 384-398 (2005).
11 McClanahan, T. & Obura, D. Sedimentation effects on shallow coral communities in Kenya. *J. Exp. Mar. Biol. Ecol.* **209**, 103-122 (1997).
12 Guest, J. *et al.* 27 years of benthic and coral community dynamics on turbid, highly urbanised reefs off Singapore. *Sci. Rep.* **6** (2016).
